# The Dental Law of Indiana as Amended in 1887

**Published:** 1887-04

**Authors:** 


### The Dental Law of Indiana as Amended in 1887.

### AN ACT

To regulate the practice of Dentistry in the State of Indiana; providing the manner of selecting a Board of Examiners; the examinations and qualifications of applicants; the issuing of certificates; the meeting of said Board; the term of office and pay; the issuing of permits by individual members of said Board; the recording of certificates by holders; prescribing penalty for the violation of the provisions of this Act; repealing the Act of March 29, 1879, and all conflicting laws; and providing for prosecutions of any violations of the Act of 1879 under the provisions thereof.

---

SECTION 1. Be it enacted by the General Assembly of the State of Indiana, that it shall be unlawful for anyone to practice dentistry in the State of Indiana at any time after thirty days from the appointment of the Board of Examiners, provided for in section two hereof without being registered according to the provisions of this act

SEC. 2. A Board of Examiners, consisting of five reputable practicing dentists, shall be appointed on the last Tuesday of June, 1887, and biennially thereafter, one by the Governor, one by the State Board of Health, and three by the Indiana State Dental Association, said Board to serve for the term of two years from the date of such appointment; and it shall be the duty of said Board to meet annually, at the time and place fixed for the meeting of said Dental Association, or oftener at the call of any three members of said Board, at such time and place as may be designated in such call. When convened the said Board shall examine all applications, issue registration certificates thereon, and also examine all applicants for certificates of qualification, and issue such certificates to all such applicants as shall pass a satisfactory examination.

SEC. 3. Any person who shall prove to the satisfaction of said Board of Examiners that he is a graduate of a Dental College duly and legally incorporated, and who shall present a diploma therefrom, and shall further show that said College is of good repute, shall be entitled to a registration certificate on the payment of a fee of one dollar to said Board.

SEC. 4. Any person who shall present to said Board of Examiners a valid certificate of qualification, issued by the Board of

Examiners under the provisions of any former law of this State, shall be entitled to a certificate of registration upon the payment of a fee of one dollar to said Board.

Sec. 5. Any person who shall file before said Board of Examiners an application under oath, and sworn to by one or more freeholders, setting forth the fact that said applicant has been engaged in the lawful practice of dentistry in this State continuously since the 29th day of May, 1879, shall be entitled to a registration certificate, on the payment of a fee of one dollar to said Board.

Sec. 6. Any person who shall desire to obtain a certificate of qualification to practice dentistry in this State, and who shall not be entitled to a registration certificate under any of the provisions of the preceding sections of the act, shall be by said Board examined in anatomy, physiology, pathology, therapeutics, chemistry, and the theory and practice of surgical and mechanical dentistry, upon the payment of a fee of five dollars to said Board, and should such examination of said applicant prove satisfactory to said Board, it shall issue to said applicant a certificate of qualification and registration.

Sec. 7. Any member of the Board of Examiners may grant a permit to practice dentistry to any person who shall file with said member his application therefor, but such permit shall only be valid until the next meeting of said Board.

Sec. 8. All certificates (except permits) issued under this act shall be signed by at least three members of said Board of Examiners, and said certificates shall have the seal of the "Indiana State Dental Association" affixed thereto. A majority of said Board shall constitute a quorum to transact business.

Sec. 9. All persons receiving certificates of registration from said Board of Examiners, or permits from any member thereof, before beginning to practice dentistry, shall present said certificate of registration, or permit, to the Recorder of the County wherein said applicant desires to practice, and the said Recorder shall record said certificate, or permit, in the miscellaneous record of his office, and said Recorder shall indorse the recording of the same on the applicant's certificate, or permit, and for his services he shall collect from each applicant the sum of twenty-five cents.

SEC. 10. Any person who shall violate any of the provisions of this act shall, upon conviction thereof, be fined not less than twenty nor more than one hundred dollars for each offense: PROVIDED, That nothing in this act shall be construed to prevent any lawfully registered surgeon or physician from extracting teeth, or performing any surgical operation in the line of his professional duties.

SEC. 11. The Board shall receive out of the fund created by this act such compensation for their services as the by-laws of said State Dental Association may provide.

SEC. 12. An act entitled "An act to regulate the practice of dentistry" approved March 29, 1879, and printed in the Revised Statutes of 1881 as chapter 47, and being sections 4249 to 4257 inclusive, be and the same is hereby repealed, together with all laws in conflict with this act: Provided, however, That all violations of the laws hereby repealed may be prosecuted under the provisions of the laws in force at the time when such offense was committed.

